# East London Medical Society and Hospital Abuse

**Published:** 1904-11-05

**Authors:** F. Harris White

**Affiliations:** Hon. Secretary, East London Medical Society


### EDITOR'S LETTER-BOX.

### EAST LONDON MEDICAL [illegible]ETY AND HOSPITAL ABUSE.

*To the Editor of* THE HOSPITAL.

DEAR SIR,—Since the appearance in the public press of the report of the quarterly meeting of the Governors of the London Hospital many comments have been made on it, and allusion has been frequently made to the East London Medical Society; so much so that the Society feels that it is necessary to clearly define its position, in order that there may be no misunderstanding in the mind of the public as to the object it is striving for.

Last May a deputation of the East London Medical Society waited on the Board of Management of the London Hospital to ask for a reform of various abuses in the management of the hospital. The abuses complained of fell under two main heads:—(1) The part payment system under which patients are expected to pay a proportion of the cost of the medicines and dressings supplied to them; and (2) insufficient scrutiny, whereby patients, who are well able to pay for treatment privately, are enabled to take advantage of a public charitable institution.

The existence of abuses was admitted by the hospital authorities and it was shown that in one important branch, the casualty department or receiving-room, the scrutiny was not merely imperfect but practically non-existent; for the addresses of patients were not asked, all comers being treated without question as to their means. The steps proposed by the hospital for the abatement of the evil have now appeared in the press, and it is principally on them that the East London Medical Society wishes to express its views.

1. "Large notices have been placed in the outpatients' department stating that a small charge is made for medicines and dressings, but no charge for advice." The part payment system, which is thus affirmed, is objected to by medical practitioners, because they find that it induces many patients who are too well off to deserve hospital relief to go to hospitals for treatment in the belief that they are paying for all they receive, and so are not receiving charity; and it also acts as a salve to the consciences of others who go to hospitals knowing that they have really no right to hospital relief. The Society holds that notices should be placed in all departments of the hospital and on the cards issued to patients that the institution is for charitable relief only.

2. "The addresses of all patients will be taken, and the books open to the inspection of any local practitioner who cares to inspect them." The registration of addresses is so obviously necessary in any inquiry as to the means of the patients, that it is amazing that it should ever have been neglected; but the inspection of the books by local practitioners is a part of that system of espionage to which the Society strongly objects.

3. "Local practitioners are invited to visit the outpatients' department, and on recognising any patient known as being unsuitable for hospital treatment, asked to communicate with the secretary." This suggestion, which invites medical men to act as detectives, was made to the deputation of the Society at the hospital, and was rejected as quite impossible. The hospital would have shown better taste had it let the suggestion drop, instead of repeating it and allowing it to go to the press.

4. "The Jewish Board of Guardians have been approached, and consented to help the hospital in making inquiries with regard to Jewish patients." The intimate acquaintance of the Jewish Board of Guardians with the Jewish poor should be a great help in eliminating unsuitable cases.

5. "The Metropolitan Provident Dispensary, which has a branch opposite the hospital, has agreed to take over any patients sent from the hospital at a reduced charge." The Society has been informed that the dispensary intends to institute a further inquiry to ascertain whether the patients are poor enough to deserve dispensary treatment. Without this the hospital plan would be most futile, for large numbers of patients now treated at the hospital are well able to pay the fees of a private practitioner, and consequently have no right to go to either the dispensary or the hospital. It is not easy to see why most of the unfit cases should not be referred direct to private practitioners, for the difference between payment for medicine and dressings at the hospital and reduced payments at the dispensary cannot be very great.

6. "The committee is in communication with the Charity Organisation Society, and it is hoped that this society will be able to advise the hospitals as to the best means for inquiring about patients, while the hospital is willing to pay £100 per annum to the society for an almoner to be stationed in the receiving rooms." This should lead to an improvement in the inquiry system and so to more efficient detection of offenders.

7. "The secretary has made arrangements to visit every patient admitted on the day after arrival, and if found the patient should not receive hospital treatment, he or she is to be discharged and asked to give a donation for the time he or she remains in the hospital." This is not an entirely practicable suggestion. In a hospital where the admissions are so numerous (sometimes, it is said, as many as 60 in a day) it is not possible for a busy man like the secretary to inquire into the circumstances of them all. By all means let any urgent case be admitted at once without inquiry, but whenever there is no urgency the admission order ought not to be given until an inquiry has shown that the case is one suitable for hospital relief. The society greatly regrets that no rule has been made that all patients whose means are sufficient to enable them to pay the fees of a private practitioner shall be refused any attendance beyond first aid. In this way a considerable economy of the hospital funds might be effected.

In conclusion the Society, while recognising the extreme difficulty of preventing abuse, wishes to point out (1) That at present advantage is taken of the lax inquiry system by a very large number of persons who ought not to receive charitable relief, and who in many instances are really wealthy; (2) That the funds supplied by the charitable are thus misappropriated, the persons for whom they are intended being often deprived of relief in consequence; (3) That the medical profession is subjected to a most unfair competition with subsidised institutions; (4) That the indiscriminate administration of relief tends to pauperise many of the recipients and to lessen habits of thrift; (5) That with more efficient means of preventing abuse the poverty of hospitals would be diminished, perhaps even abolished; and (6) That the hospital authorities must not forget in delegating their duties of scrutiny, that *they* are primarily responsible to the charitable public for the proper expenditure of the monies subscribed.

Yours faithfully,

F. Harris White,

Hon. Secretary, East London Medical Society.

